# The Aftermath of Pulmonary Embolism: Are Residual Thrombi Clinically Significant?

**DOI:** 10.3390/diagnostics15111348

**Published:** 2025-05-27

**Authors:** Irina Pocienė, Edvardas Danila

**Affiliations:** 1 Centre of Pulmonology and Allergology, Vilnius University Hospital Santaros Klinikos, LT-08406 Vilnius, Lithuania; edvardas.danila@santa.lt; 2 Clinic of Chest Diseases, Immunology and Allergology, Faculty of Medicine, Institute of Clinical Medicine, Vilnius University, LT-03101 Vilnius, Lithuania

**Keywords:** pulmonary embolism, residual pulmonary vascular obstruction, long-term outcomes

## Abstract

**Background:** Following acute pulmonary embolism (PE), disease outcomes vary among patients. Complete recovery occurs in some cases, while others may experience persistent long-term symptoms, disease recurrence, or progression to chronic thromboembolic pulmonary hypertension (CTEPH). The exact reasons behind incomplete recovery and different outcomes are still not well established. This review aims to present the existing data regarding the clinical significance of residual thrombi after acute PE, particularly in the context of disease recurrence, the development of CTEPH, or persistent symptoms and functional limitations. **Methods:** Original articles, systematic reviews, and meta-analyses relevant to the topic are reviewed. **Results:** Incomplete thrombus resolution after acute PE is quite common, with studies showing that it affects one-fourth to one-third of PE patients, despite receiving optimal anticoagulant treatment. It has been shown that residual thrombi after acute PE play a role in the risk of PE recurrence. However, there is still no standardized method to differentiate disease recurrence from residual thrombi in pulmonary imaging studies, particularly in cases where no follow-up scans and different imaging techniques are used for thrombi detection. Residual vascular obstruction is necessary for the development of CTEPH. Evidence suggests that the extent of residual thrombi contributes to a higher risk of CTEPH. Still, there is a need to standardize both the timing of residual thrombi assessment and the evaluation of their distribution, in relation to the development of CTEPH. The significance of residual thrombi for persistent symptoms and functional limitation remains debatable. Research indicates that nearly half of patients experience long-term symptoms after acute PE. Still, it is believed that these symptoms are not necessarily caused only by residual thrombi, but rather by the worsening of other comorbid conditions. **Conclusions:** Studies show that residual thrombi after acute PE are significant for PE outcomes. It may be beneficial to consider evaluating residual pulmonary vascular obstruction when treating patients after acute PE to optimize the duration of anticoagulant therapy and improve patient outcomes.

## 1. Introduction

Venous thromboembolism (VTE), clinically manifesting as either PE or deep vein thrombosis (DVT), represents the third most common acute cardiovascular disorder globally [1]. PE incidence rates range from 39 to 115 individuals per 100,000 population [2]. As a highly prevalent disease, PE affects both sexes and impacts individuals across all age groups worldwide, due to variable risk factors [3].

Advancements in diagnostics have significantly improved the short-term prognosis of PE. Clinical probability estimation with age-adjusted D-dimer testing has demonstrated potential in safely excluding PE [4]. Innovations in CT imaging, such as dual-energy computed tomography, have markedly enhanced diagnostic accuracy for acute PE [5]. Risk stratification in PE patients also enables clinicians to adjust treatment strategies for each individual [6]. These advancements have contributed to a notable increase in the detection of new PE cases and a reduction in PE-related mortality, from 12.8 to 6.5 deaths per 100,000 population over the past few decades [7]. However, this progress has introduced new challenges in clinical practice. Progress in the short-term management of PE shifted the perception of the disease, recognizing it not only as an acute event with short-term treatment but also as a potentially chronic condition requiring long-term treatment, since the disease does not resolve upon hospital discharge. Various studies indicate that 50–75% of patients experience complete thrombus resolution after 3 to 6 months of anticoagulation treatment [8,9]. In others, PE may persist long after the acute episode [10,11,12].

The significance of incomplete thrombus resolution after an acute event on PE outcomes remains unclear. The fate of residual thrombi can vary—they may persist without causing serious clinical consequences (chronic PE), lead to recurrent PE, or result in CTEPH [13]. Chronic PE is diagnosed in approximately ~25–30% of patients after acute PE [9,14]. The recurrence rate in the first year after treatment discontinuation is generally reported to be up to 10% [12]. The prevalence of CTEPH in the literature ranges from 0.5% to 3.8% of patients who survive the acute phase of PE [11,15].

Evidence suggests that residual thrombi are associated with an increased risk of PE recurrence [8,16]. Furthermore, despite CTEPH being a rare condition, residual thrombi serve as a necessary precursor for its development [11]. Its clinical significance in contributing to long-term symptoms, such as persistent dyspnea, reduced exercise capacity, or diminished quality of life, remains controversial [17].

Empirical experience in our tertiary referral University Center of Pulmonology and Allergology suggests that guiding the duration of PE treatment based on residual thrombi would be valuable in preventing treatment complications and avoiding long-term adverse consequences. Therefore, in this comprehensive review, we will discuss the current data, highlighting the importance of residual thrombi in PE outcomes. At first, the pathophysiological mechanisms underlying thrombus dissolution, covering both normal and pathological pathways, will be analyzed. The timing of possible thrombi resolution, the rates of residual thrombi following acute PE, and the potential risk factors associated with incomplete thrombus resolution will also be discussed. Then, the clinical significance of residual thrombi following acute PE, emphasizing their impact on long-term symptoms, patient quality of life, the risk of recurrent PE, and the development of chronic CTEPH, will be explored.

## 2. Materials and Methods

### 2.1. Inclusion and Exclusion Criteria

Original articles, systematic reviews, and meta-analyses, written in English, were included. Studies were eligible if the diagnosis of first-time acute PE was confirmed either by CTPA or V/Q scintigraphy, and if at least one imaging follow-up was performed after discharge (not earlier than three months) to assess for residual pulmonary vascular obstruction (RPVO). Patients in the selected studies must have had received a minimum of three months of anticoagulation therapy. Studies that investigated the clinical significance of residual thrombi after PE in relation to long-term symptoms, quality of life, recurrent PE, or the development of chronic thromboembolic pulmonary hypertension (CTEPH) were included.

The exclusion criteria included abstracts, case reports, and letters. Studies involving recurrent PE ot those using thrombus evaluation methods other than CTPA or pulmonary scintigraphy were excluded.

### 2.2. Information Sources and Search Strategy

A comprehensive literature search was conducted on the Web of Science, PubMed, and Google Scholar to identify relevant original articles, systematic reviews, and meta-analyses regarding residual thrombi after acute PE. The search strategy involved the utilization of both MeSH terms and free keywords. The following search terms were used: “residual pulmonary vascular obstruction AND pulmonary embolism”, “residual thrombi AND pulmonary embolism”, “residual clot AND pulmonary embolism” as well as “long-term symptoms”, “quality of life”, “chronic thromboembolic pulmonary hypertension (CTEPH)”, “recurrent pulmonary embolism”.

### 2.3. Study Selection

**Scheme 1:**
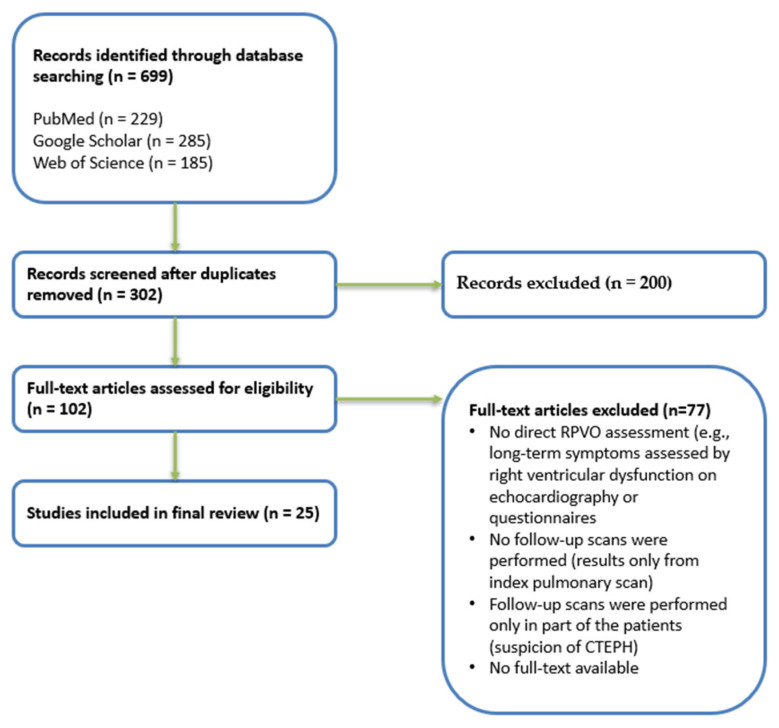
Selection of studies for inclusion in the review.

The titles and abstracts of the identified articles were initially screened to assess their relevance to the topic. A final selection was made based on inclusion and exclusion criteria after a full-text reading of the studies (Scheme 1).

This review does not aim to cover or summarize all existing literature systematically. The focus is on providing a narrative overview rather than a full systematic review. Therefore, some studies already included in recent systematic reviews or meta-analyses covered in this article were not analyzed in detail. A few excluded studies are also discussed to highlight the lack of original research on specific aspects.

## 3. Definitions in Pulmonary Embolism: Residual Thrombus, Chronic PE, PE Recurrence, and CTEPH

### 3.1. Residual Thrombus

Residual thrombus refers to a persistent intraluminal thrombotic material identified on follow-up imaging after 3 to 6 months following an acute PE, despite a continuous course of therapeutic anticoagulation. This includes treatment with direct oral anticoagulants (DOACs), warfarin (maintaining a target INR of 2–3), or low-molecular-weight heparins administered by current clinical guidelines and patient adherence [6,18]. Sometimes, distinguishing residual thrombi from chronic thromboembolic changes and pre-existing CTEPH present before the acute PE can be challenging. According to the literature, CTEPH is frequently misclassified as acute PE [19]. However, imaging techniques, such as computed tomography pulmonary angiography (CTPA) and dual-energy computed tomography, can help differentiate between acute, resolving, or organised thrombi in pulmonary arteries. Persistent abnormalities in ventilation/perfusion (V/Q) and V/Q single photon emission tomography (SPECT) scanning can indicate chronic PE [20]. CTPA can also help to determine if signs of pulmonary hypertension (PH) in acute PE are acute or chronic (based on right ventricular enlargement or hypertrophy, the presence of collateral vessels or mosaic perfusion, etc.) [21].

### 3.2. Chronic Pulmonary Embolism

The presence of RVPO on imaging after ≥3 months of anticoagulation with no evidence of pulmonary hypertension at rest is referred to as chronic PE [22].

### 3.3. Pulmonary Embolism Recurrence

This is a new episode of PE after the initial event, typically presenting with acute symptoms, such as sudden shortness of breath, chest pain, rapid heart rate, and others. Sometimes, distinguishing between a true PE recurrence and the persistence of residual thrombi can be challenging, especially in the absence of baseline images and follow-up scans, or when different assessment methods are used [23]. In cases where imaging results are inconclusive, clinical assessment and follow-up are crucial [24]. As mentioned previously, recurrent PE typically presents with new or worsening symptoms that other causes cannot explain. In contrast, residual thrombus is often asymptomatic or associated with persistent, non-progressive symptoms. Laboratory findings can help in differentiation: an elevated D-dimer level, particularly if previously normalized, may indicate recurrence. Elevated cardiac biomarkers, such as troponin and brain natriuretic peptide (BNP), may further support the diagnosis of PE reccurence [24]. Imaging findings, such as CTPA, can also help distinguish between acute and chronic PE. In acute PE, embolus may totally or partially occlude the vessel. In case of partial occlusion, it typically appears as a central filling defect within an otherwise contrast-enhanced vessel. Affected vessels may appear slightly enlarged. Acute PE may also be associated with pulmonary infarction and signs of right heart strain [21].

### 3.4. Chronic Thromboembolic Pulmonary Hypertension

CTEPH is the occlusion of pulmonary arteries by fibrotic material, leading to increased pulmonary vascular resistance and progressive right heart failure, which may or may not present with symptoms [25]. The diagnosis of CTEPH is based on findings of right heart catheterisation obtained after at least 3 months of adequate anticoagulation: mean pulmonary arterial pressure (mPAP) ≥ 20 mmHg with pulmonary artery wedge pressure (PAWP) ≤ 15 mmHg, pulmonary vascular resistance (PVR) > 2 Wood units and mismatched perfusion defects on lung scan. Specific diagnostic signs for CTEPH seen by multidetector CTPA, MRI, or conventional pulmonary angiography support the diagnosis [26]. Pulmonary endarterectomy (PEA) is the choice of treatment in appropriately selected patients. Balloon pulmonary angioplasty (BPA) is recommended for inoperable CTEPH patients or patients with residual PH after PEA. PH medications (Riociguat, other PH medications approved in select regions) are given for CTEPH patients who are not eligible for interventional treatments. Lifelong anticoagulation is recommended for all patients with CTEPH [27].

## 4. Pathophysiology of Thrombus Resolution

Following the initial impact of thromboembolism, a thrombus’s natural resolution is crucial for restoring normal vascular flow. Several mechanisms, including endogenous fibrinolysis, angiogenesis, and inflammation, play a key role in determining the outcome of a thromboembolus [28]. Among these, fibrinolysis is the most important one. It starts immediately after thrombus formation and helps to dissolve fibrin networks within the clot, enabling the complete resolution of thromboemboli of varying sizes [29]. Inflammatory cells, such as neutrophil granulocytes, are the predominant leukocytes in thrombi during the early stages of clot resorption. Monocytes, which infiltrate the thrombus around day 5 and gradually increase in number for up to three weeks, secrete proteases, chemokines, and growth factors essential for the resolution process [30]. Angiogenesis helps restore blood flow to the affected area and clear the thrombus. It is driven by pro-angiogenic factors, such as vascular endothelial growth factor (VEGF) and fibroblast growth factor (FGF), which are released in response to tissue hypoxia and inflammation [31].

Lungs are remarkable in clearing thromboemboli, partly because the high blood flow exposes thrombi to more plasminogen and because the cells lining the pulmonary artery might be better at breaking down clots than those in other veins [32]. Under normal conditions, when all mechanisms function in coordination, small thrombi begin to resolve within 5–7 days following an acute event, with most thrombi resolving within 2–3 months [33].

## 5. Risk Factors and Mechanisms of Incomplete Thrombus Resolution

As discussed earlier, following PE thrombi in the pulmonary arteries are either resolved by fibrinolysis or are remodeled and organized into fibrotic tissue [29]. The exact reason why some pulmonary emboli do not completely resolve remains unclear, not only because it is likely that there are many contributing factors, but also because the analysis of potential mechanisms underlying incomplete thrombus resolution is complicated by the lack of reliable models, which will also be briefly covered later [34].

Experimental studies using rodent models to evaluate the pathogenesis of PE are widely accepted and commonly used. However, challenges arise when trying to model thrombi resolution and the development of CTEPH due to the high fibrinolytic activity of rodent blood plasma [35]. Currently, there are no optimal solutions for these modeling difficulties. Autopsy-based studies typically focus on analyzing the age of thrombi and identifying thrombi at various stages of resolution. However, they provide limited information about the mechanisms of thrombi resolution [36].

It is hypothesized that residual pulmonary vascular obstruction (RPVO) is associated with impaired fibrinolysis due to intrinsic patient factors, thrombus characteristics, or induced hypercoagulability [37]. Abnormal thrombus remodeling and scar tissue formation, resulting from the intense inflammatory response during acute PE, along with defective angiogenesis, neovascularization, and smooth muscle hypertrophy, also play a role in RPVO [38]. Incomplete thrombus resolution likely results from a complex interplay of multiple different factors.

**Figure 1:**
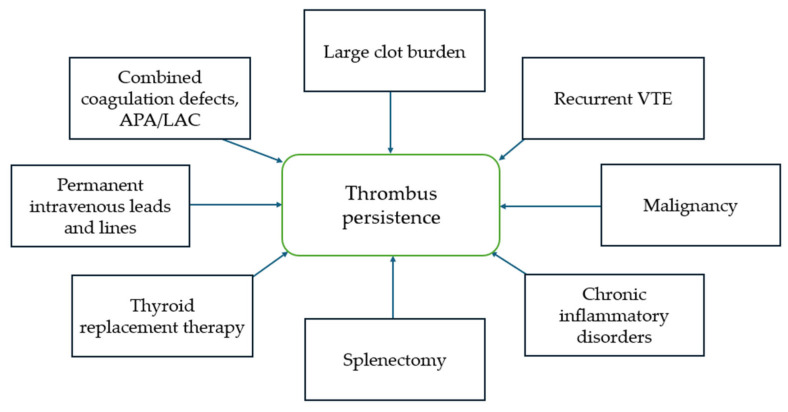
Risk factors for thrombus persistence. APA—antiphospholipid antibodies; LAC—lupus anticoagulant; VTE—venous thromboembolism.

Without a comprehensive understanding of the pathogenesis of unresolving thrombi, clinical conditions that increase the probability of thrombus persistence are distinguished (Figure 1). These conditions are associated with hypercoagulable states, chronic inflammation, disturbance in various hemostatic and fibrinolytic parameters, and coagulation defects [31].

## 6. Timing of Thrombus Resolution and Prevalence of Residual Thrombi After Acute PE

The timing of thrombus resolution and the prevalence of residual thrombi after acute PE vary between studies. Differences in clot burden observed in patients after acute PE may be influenced by several factors, discussed below [8,9,14,23,39,40,41,42,43].

Published results of the extent of RPVO in studies depend on the method used to assess residual thrombi during follow-up visits, whether using pulmonary perfusion (Q), ventilation/perfusion (V/Q) scans, or CTPA scans. The rate of RPVO is found to be lower in CTPA scans compared to perfusion lung scans [40,42]. Due to greater sensitivity and ability to minimize radiation exposure, many studies use lung perfusion scans to assess RPVO [14,39,40]. However, it is important to consider that non-embolic pulmonary diseases may increase RPVO by omitting the ventilation scan.

Perfusion defects are also defined differently between studies [9,42]. Some define pulmonary vascular obstruction as any perfusion defect detected. In contrast, others use a cut-off value of 10%, which corresponds to the amputation of at least two lung segmental arteries [44].

**Figure 2:**
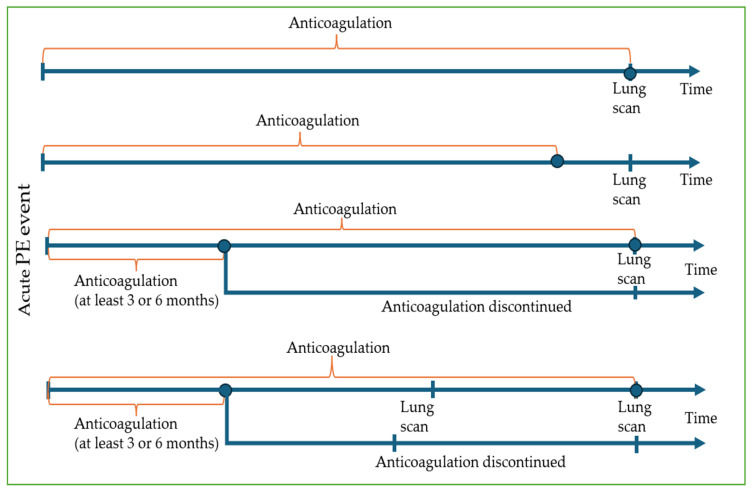
Schematic representation of differences in study designs.

Differences in study designs (summarized in Figure 2) may also influence the results of thrombus resolution time and prevalence following acute PE. Some studies assess RPVO during anticoagulant discontinuation [23,39], while others perform lung scans after a certain period following treatment discontinuation [14,43]. There are also studies where anticoagulation is administered for at least 3 or 6 months after acute PE, with some patients still on anticoagulants and others having already discontinued treatment at the time of RPVO assessment [41,42]. In these studies, individual risk factors typically guide the treatment duration.

In most studies, patients with PE are initially treated with LMWH. In selected high-risk cases, thrombolytic therapy is administered by current clinical guidelines. This initial phase is typically followed by long-term anticoagulation with vitamin K antagonists (VKAs), most commonly warfarin [9,14,40]. There is a lack of information about the quality control of the treatment, leaving uncertainty as to whether it might have influenced RPVO at follow-up visits.

While VKAs have long been used for PE treatment, DOACs (factor Xa inhibitors apixaban, rivaroxaban, enoxaban, and trombin inhibitor dabigatran) have become the preferred option in many international guidelines due to their predictable bioavailability and pharmacokinetics [6,45,46]. DOACs can be given at fixed doses, do not require routine INR monitoring, have fewer dietary and drug interactions, and are associated with comparable or lower risk of bleeding than VKAs [6,47]. In cases where extended anticoagulation is indicated, apixaban and rivaroxaban may be continued at lower doses, potentially minimizing the risk of adverse events [48,49].

In the context of residual thrombus, direct comparative evidence between VKA and direct oral anticoagulants (DOACs) is limited. A meta-analysis by Nick van Es et al. found that DOACs had similar efficacy to VKAs in preventing recurrent VTE [50]. However, this analysis did not specifically evaluate residual thrombus resolution in PE. Most studies on RVPO either include only warfarin or report outcomes for both VKAs and DOACs without directly comparing their impact to residual thrombi [41,42,43]. Therefore, further research is needed to examine the effects of novel oral anticoagulants (NOACs) on RPVO.

It should also be noted that some studies assessing the prevalence of residual thrombus do not include patients with risk factors associated with a longer thrombus resolution time and chronic PE. For example, in the study by Jervan et al. [43], one of the exclusion criteria was active malignancy.

Despite differences in study design, methods of RPVO assessment, and treatment duration, studies conducted in the last few decades indicate that one-fourth to one-third of patients still experience RPVO on pulmonary scans up to one year following acute pulmonary embolism (see Table 1 for results). Some studies have shown residual thrombi in more than half of patients during follow-up lung scans [8,23]. Therefore, thrombi remain in the pulmonary arteries long after acute PE in many patients.

As evidence shows, thrombi resolve more slowly than previously thought, and their resolution depends on risk factors. Treatment duration for PE is increasingly individualized. The ESC guidelines for PE diagnosis and treatment recommend not only indefinite treatment duration for patients with antiphospholipid syndrome or active cancer, but also recommends considering extended-duration therapy for patients when there is no identifiable risk factor, for those with a persistent risk factor, or even in cases where a minor transient risk factor was present [6].

## 7. Clinical Significance of Residual Clots

### 7.1. Residual Thrombi and PE Recurrence

Anticoagulant treatment effectively reduces the risk of recurrence in VTE. However, this benefit disappears once treatment is discontinued [51,52]. The risk of PE recurrence depends on individual patient risk factors (see Table 2) [6]. The risk of recurrent VTE after the first unprovoked event is 10% within the first year after discontinuation of anticoagulation and rises to 25% within five years [12]. The risk of another PE episode is three times higher in patients whose initial acute event was PE, compared to those with DVT [48,53]. Given the clinical importance of recurrent PE, it is crucial to determine whether residual thrombi following an acute PE episode have prognostic value in predicting disease recurrence.

Over the past few decades, studies have been conducted—mainly retrospective and a few prospective cohort studies—analyzing the risk of RPVO on recurrent VTE [8,54,55]. Since different results have been obtained from these studies, several meta-analyses have emerged in recent years summarizing the available data [56,57].

In 2019, Becattini et al. published a meta-analysis aimed at evaluating the prognostic value of RPVO, as assessed by either CTPA or lung perfusion scan, on the risk of recurrent VTE and PE in patients treated with anticoagulants for acute PE [56]. In total, 16 studies involving 3472 patients were analyzed. RPVO was associated with an increased risk of recurrent VTE (odds ratio [OR] 2.22; 95% confidence interval [CI] 1.61–3.05), but this association was statistically significant only when lung perfusion scans detected RPVO, and not by CTPA. Also, RVPO was associated with recurrent PE (7 studies; 1801 patients; OR 2.98; 95% CI 2.00–4.44).

In this meta-analysis by Becattini et al., a comprehensive search of databases by multiple authors was performed, ensuring an in-depth review of available studies analyzing RPVO and recurrent VTE. It includes sensitivity analysis on various parameters, such as methods of RPVO evaluation, the timing of RPVO evaluation (at least 3 months after acute PE), and symptomatic vs. asymptomatic recurrence of VTE. Heterogeneity between studies was also evaluated using Cochran’s X^2^ and the I^2^ tests. Potential limitations that may influence the results include differences in the cut-off point for RPVO between studies, the limited accuracy of currently available criteria for diagnosing VTE, particularly in patients with residual thrombi, and the smaller number of studies assessing RPVO by CT scan, which may limit the statistical power of the results in this subgroup.

In 2023, Robin et al. published a systematic review and meta-analysis of individual patient data on RPVO and VTE recurrence [57]. It includes four studies with 809 patients who received anticoagulant therapy for at least 3 months (median 6.6 months) after PE and underwent planar lung scans at the time of treatment discontinuation. RPVO was present in 50.3% of patients after a median of 6.2 months after acute PE. During a median follow-up of 26.2 months, 114 patients (14.1%) had recurrent VTE. The annual risk of recurrent VTE was 5.8% (CI 4.4–7.2) in participants with RPVO < 5%, compared to 11.7% (CI 9.5–13.8) in those with RPVO ≥ 5%. RPVO was a predictor of recurrent VTE in both provoked and unprovoked PE.

The strength of the Robin et al. study is that individual patient data from included studies were obtained [57]. This helped to study specific subgroups of interest and determine optimal cut-off values for RPVO.

There is an association between RPVO and an increased risk of VTE (PE or DVT) recurrence after initial anticoagulant treatment. However, currently, there are no well-established guidelines for distinguishing between recurrent PE and chronic unresolved thrombi on lung imaging, particularly in the absence of follow-up scans using the same imaging technique. Diagnosis is often challenging even in cases where the recurrence of PE is symptomatic. According to current data, RPVO evaluation might be beneficial for guiding anticoagulant treatment duration and secondary VTE prevention [58].

### 7.2. Residual Thrombi and CTEPH

CTEPH is a rare complication of PE, associated with significant morbidity and mortality [25]. The incidence of CTEPH differs between studies. It is estimated to be around 0.5–3.8% [11,54]. It is known that RPVO is necessary, but not always sufficient, to develop CTEPH. Clinicians face the challenge of identifying patients at an increased risk of CTEPH. There is still a lack of studies analyzing the clinical significance of RPVO extent and other possible risk factors associated with CTEPH development [8,59,60,61].

In 2017, Chopard et al. conducted a prospective multicenter study involving 241 patients following acute PE, who underwent at least two V/Q scans at discharge and 3 months after acute PE [59]. The study found that RPVO > 15% at 3 months and the difference in RPVO between discharge and 3 months after index PE ≤ 37.5% were significantly associated with the occurrence of a combined endpoint (consisting of all-cause mortality, recurrent VTE, CTEPH, heart failure, and hospitalization for cardiac causes at 5 years). However, when examining CTEPH development after 5 years, the incidence of RPVO ≥ 15% was higher, though it did not reach statistical significance.

This study, published by Chopard et al., has an extended follow-up duration, providing robust data on long-term outcomes [59]. However, the sample size of the study is relatively small. The study results could also be influenced by the duration of anticoagulation therapy, as the decision to continue treatment after 3 months was left to the physician’s discretion. Also, the significance of RPVO in CTEPH development was observed only in the context of the combined endpoint.

In a randomized, double-blind, multicenter study, involving 371 patients after the first unprovoked PE, RPVO and systolic pulmonary artery pressure (sPAP) were identified as the predictors for CTEPH diagnosis [60]. It was found that RPVO > 45% with a hazard ratio (HR) of 33 (95% CI 1.64–667, *p* = 0.02) at PE diagnosis and RPVO >14% at 6 months after acute PE (HR) of 63.9 [95% CI 3.11–1310, *p* < 0.01] were related with the development of CTEPH for 8 years follow-up period. This was the first prospective study on the incidence of CTEPH in patients with the first unprovoked PE, involving a homogeneous and well-defined population. The follow-up of the patients in this study was long (8 years), with a low number of patients lost during that time. A potential limitation that may influence the study’s statistical power is the small sample size of CTEPH cases, with only nine patients diagnosed during the follow-up period.

There is also a retrospective study published in 2023, where clot burden was scored through the Qanadli method (QS) on the initial CTPA (at the time of diagnosis). Over a one-year follow-up period, 22 (6.8%) out of 475 patients were diagnosed with CTEPH. The QS from the initial CT was directly associated with CTEPH, and a cut-off point of 14.5 (43.5%) was predictive for CTEPH in men [62]. This study analyzes only the significance of initial RPVO for CTEPH, and V/Q scans during follow-up were performed only in symptomatic patients. The extent of clot burden found in V/Q scans of patients diagnosed with CTEPH is also not specified.

In 2024, Liu et al. proposed a clinical prediction model for the early identification of chronic PE [63]. It included 464 patients with acute PE, who were followed for at least 3 months and then divided into chronic PE and non-chronic PE groups based on symptoms and RPVO on CT angiography or V/Q scans. Of 130 (28%) patients with chronic PE, 17% developed CTEPH. Time from symptoms onset to PE diagnosis ≥15 days, recurrent PE, RVD, central embolus, and RPVO >10% were identified as independent predictors of CTEPD, which may lead to the development of CTEPH. A prediction model with a C-index of 0.895 (95% CI 0.863–0.927) was established for high-risk patients. The study was internally validated and utilized advanced predictive modeling. However, the sample size (140 patients in the validation cohort) may limit the analysis of risk factors. Also, V/Q scans or CTPA were performed only in symptomatic patients during follow-up. It is not specified whether there were patients with RPVO who exhibited no symptoms.

In summary, the extent of RPVO influences the development of CTEPH. However, there is still a lack of evidence on the optimal timing to assess RPVO. Additional studies, including systematic reviews and meta-analyses, are needed to clarify the extent of RPVO that is clinically significant for the development of CTEPH, as this value varies between studies.

### 7.3. Residual Thrombi and Long-Term Symptoms, Quality of Life

It is increasingly evident that many patients experience long-term consequences following acute PE. Nearly half of the patients experience chronic symptoms and functional limitation [17,61]. Health-related quality of life is also lower in patients after acute PE [57]. Recently, it was proposed to refer to this phenomenon of long-lasting symptoms and functional limitations as the “post-pulmonary embolism syndrome” [61].

Unfortunately, it is still unclear whether persistent symptoms, functional limitations, or worsened quality of life after acute PE are caused by PE itself or other factors, such as the worsening of comorbid diseases. In the past decade, numerous studies focusing on long-term symptoms and functional limitations after PE were conducted. However, most studies do not assess residual thrombi after the acute PE period [61,64]. Only a few of them analyze whether these symptoms could be linked to residual clot burden detected on CT or V/Q scan [59,65,66].

Recently, a systematic review and meta-analysis were published summarizing the existing data on the impact of residual perfusion defects on long-term symptoms [67]. In total, 12 studies, involving 1888 patients, were included. After a median of 6 months, 34% of patients had RPVO, and 48% were symptomatic compared to 34% of patients without RPVO. RPVO was associated with long-term symptoms (OR 2.15, 95% CI 1.66–2.78; I^2^ = 0%, τ = 0). However, no association was found between RPVO and quality of life or cardiopulmonary exercise test parameters.

In summary, patients with RPVO have an increased likelihood of experiencing persistent symptoms after PE. However, many patients with RPVO remain asymptomatic, while some without RPVO may still develop prolonged symptoms. Given that RPVO does not clearly correlate with quality of life or functional limitation, it suggests that additional factors contribute to dyspnea in these patients.

## 8. Conclusions

The timing of complete thrombus resolution after acute PE varies between patients. It should be emphasized that chronic residual pulmonary vascular obstruction after acute PE is also common. Residual thrombi play a role in the risk of PE recurrence. However, the issue of differential diagnosis remains unresolved, and there are no clear recommendations on distinguishing PE recurrence from residual thrombosis after acute PE, sometimes even in the cases of symptomatic PE recurrence. It seems that the development of CTEPH depends on the extent of the thrombus in lung scans, along with other factors. Further clarification is needed regarding the timing of lung scans and the exact extent of residual pulmonary vascular obstruction related to a higher risk of CTEPH. Symptomatic and functional recovery after PE depends on many factors, possibly including thrombus resolution.

Based on existing data, performing follow-up scans after acute PE and using residual thrombi to guide treatment to improve patient outcomes may be beneficial.

## Figures and Tables

**Table 1:** Prevalence of residual thrombi after PE according to studies.

Year	Study	Number of Patients	Time for Assessment	Imaging Techniques	Prevalence of Residual Abnormalities	Treatment	Treatment Duration
2006	Miniati et al. [39]	235	1 year	Q lung scan	34.9%	VKA (91.9%)	1 year
2010	Sanchez et al. [9]	254	6–12 months	V/Q lung scan (>10% RPVO)	29%	VKA	6 months (median)
2011	Cosmi et al. [40]	173	9 months	Q lung scan MDCT scan	28% 15%	VKA	9 months (median)
2013	Poli et al. [14]	235	11 months	Q lung scan	26%	VKA	12 months (median) Indefinite treatment time in 41% of patients
2016	Planquette et al. [41]	321	9 months	V/Q lung scan	19%	VKA	6.5 months
2017	Pesavento et al. [8]	647	6 months	Q lung scan	50.1%	VKA	at least 6 months
2018	K.A. Ma et al. [42]	82 73	1 year	MDCT Q lung scan (>0% RPVO)	15.9% 41.1%	VKA	5.7 (mean)
2022	Yoshihisa Nakano et al. [23]	43	1 year	MDCT	79%	DOAC	95% of patients are still on anticoagulation at 1 year
2023	Øyvind Jervan et al. [43]	286	6–72 months	V/Q scan	25.2%	NA	8 months (no RPVO) 9 months (with RPVO)

Q—perfusion scan, V/Q—ventilation/perfusion scan, MDCT—multidetector computed tomography, RPVO—residual pulmonary vascular obstruction, VKA—vitamin K antagonists, DOAC—direct oral anticoagulants, NA—not available.

**Table 2:** Risk factors for recurrent venous thromboembolism.

Risk for Recurrence	Risk Factors
Low (<3%)	Major transient or reversible factors (e.g., surgery with general anaesthesia for >30 min.; trauma with fractures).
Intermediate (3–8%)	Transient or reversible factors (e.g., admission to hospital for <3 days with an acute illness; oestrogen therapy/contraception; leg injury (without fracture) associated with reduced mobility ≥3 days; long-haul flight). Non-malignant persistent risk factors (e.g., active autoimmune disease; inflammatory bowel disease). No identifiable risk factor
High (>8%)	Active cancer; previous VTE in the absence of a major transient or reversible factor; antiphospholipid antibody syndrome

## Data Availability

Not applicable.

## Abbreviations

The following abbreviations are used in this manuscript:

PE	Pulmonary embolism
CTEPH	Chronic thromboembolic pulmonary hypertension
VTE	Venous thromboembolism
DVT	Deep vein thrombosis
RPVO	Residual pulmonary vascular obstruction
VEGF	Vascular endothelial growth factor
FGR	Fibroblast growth factor
APA	Antiphospholipid antibodies
LAC	Lupus anticoagulant
Q	Perfusion
V/Q	Ventilation/Perfusion
CTPA	Computed tomography pulmonary angiography
MDCT	Multidetector computed tomography
NOACs	Novel oral anticoagulants
DOACs	Direct oral anticoagulants
VKA	Vitamin K antagonists
NA	Not available
RVD	Right ventricular dysfunction
sPAP	Systolic pulmonary artery pressure

## References

[1] Raskob G.E., Angchaisuksiri P., Blanco A.N., Buller H., Gallus A., Hunt B.J., Hylek E.M., Kakkar A., Konstantinides S.V., McCumber M. (2014). Thrombosis: A major contributor to global disease burden. Arterioscler. Thromb. Vasc. Biol..

[2] Wendelboe A.M., Raskob G.E. (2016). Global burden of thrombosis: Epidemiologic aspects. Circ. Res..

[3] Di Nisio M., van Es N., Büller H.R. (2016). Deep vein thrombosis and pulmonary embolism. Lancet.

[4] Den Exter P.L., van der Hulle T., Klok F.A., Huisman M.V. (2014). Advances in the diagnosis and management of acute pulmonary embolism. Thromb. Res..

[5] Hong Y.J., Shim J., Lee S.M., Im D.J., Hur J. (2021). Dual-Energy CT for Pulmonary Embolism: Current and Evolving Clinical Applications. Korean J. Radiol..

[6] Konstantinides S.V., Meyer G., Becattini C., Bueno H., Geersing G.J., Harjola V.P., Huisman M.V., Humbert M., Jennings C.S., Jiménez D. (2020). 2019 ESC Guidelines for the diagnosis and management of acute pulmonary embolism developed in collaboration with the European Respiratory Society (ERS). Eur. Heart J..

[7] Barco S., Mahmoudpour S.H., Valerio L., Klok F.A., Münzel T., Middeldorp S., Ageno W., Cohen A.T., Hunt B.J., Konstantinides S.V. (2020). Trends in mortality related to pulmonary embolism in the European Region, 2000–2015: Analysis of vital registration data from the WHO Mortality Database. Lancet Respir. Med..

[8] Pesavento R., Filippi L., Palla A., Visonà A., Bova C., Marzolo M., Porro F., Villalta S., Ciammaichella M., Bucherini E. (2017). Impact of residual pulmonary obstruction on the long-term outcome of patients with pulmonary embolism. Eur. Respir. J..

[9] Sanchez O., Helley D., Couchon S., Roux A., Delaval A., Trinquart L., Collignon M.A., Fischer A.M., Meyer G. (2010). Perfusion defects after pulmonary embolism: Risk factors and clinical significance. J. Thromb. Haemost..

[10] Klok F.A., Tijmensen J.E., Haeck M.L., van Kralingen K.W., Huisman M.V. (2008). Persistent dyspnea complaints at long-term follow-up after an episode of acute pulmonary embolism: Results of a questionnaire. Eur. J. Intern. Med..

[11] Pengo V., Lensing A.W., Prins M.H., Marchiori A., Davidson B.L., Tiozzo F., Albanese P., Biasiolo A., Pegoraro C., Iliceto S. (2004). Incidence of chronic thromboembolic pulmonary hypertension after pulmonary embolism. N. Engl. J. Med..

[12] Khan F., Tritschler T., Kimpton M., Wells P.S., Kearon C., Weitz J.I., Büller H.R., Raskob G.E., Ageno W., Couturaud F. (2021). Long-term risk of recurrent venous thromboembolism among patients receiving extended oral anticoagulant therapy for first unprovoked venous thromboembolism: A systematic review and meta-analysis. J. Thromb. Haemost..

[13] Aghayev A., Furlan A., Patil A., Gumus S., Jeon K.N., Park B., Bae K.T. (2013). The rate of resolution of clot burden measured by pulmonary CT angiography in patients with acute pulmonary embolism. AJR Am. J. Roentgenol..

[14] Poli D., Cenci C., Antonucci E., Grifoni E., Arcangeli C., Prisco D., Abbate R., Miniati M., Poli D. (2013). Risk of recurrence in patients with pulmonary embolism: Predictive role of D-dimer and of residual perfusion defects on lung scintigraphy. Thromb. Haemost..

[15] Valerio L., Mavromanoli A.C., Barco S., Abele C., Becker D., Bruch L., Ewert R., Faehling M., Fistera D., Gerhardt F. (2022). Chronic thromboembolic pulmonary hypertension and impairment after pulmonary embolism: The FOCUS study. Eur. Heart J..

[16] Aranda C., Gonzalez P., Gagliardi L., Peralta L., Jimenez A. (2021). Prognostic factors of clot resolution on follow-up computed tomography angiography and recurrence after a first acute pulmonary embolism. Clin. Respir. J..

[17] Kahn S.R., Hirsch A.M., Akaberi A., Hernandez P., Anderson D.R., Wells P.S., Rodger M.A., Solymoss S., Kovacs M.J., Rudski L. (2017). Functional and Exercise Limitations After a First Episode of Pulmonary Embolism: Results of the ELOPE Prospective Cohort Study. Chest.

[18] Wan T., Rodger M., Zeng W., Robin P., Righini M., Kovacs M.J., Tan M., Carrier M., Kahn S.R., Wells P.S. (2018). Residual pulmonary embolism as a predictor for recurrence after a first unprovoked episode: Results from the REVERSE cohort study. Thromb. Res..

[19] Guérin L., Couturaud F., Parent F., Revel M.P., Gillaizeau F., Planquette B., Pontal D., Guégan M., Simonneau G., Meyer G. (2014). Prevalence of chronic thromboembolic pulmonary hypertension after acute pulmonary embolism. Prevalence of CTEPH after pulmonary embolism. Thromb. Haemost..

[20] Ruggiero A., Screaton N.J. (2017). Imaging of acute and chronic thromboembolic disease: State of the art. Clin. Radiol..

[21] Muscogiuri E., De Wever W., Gopalan D. (2024). Multimodality imaging of acute and chronic pulmonary thromboembolic disease. Breathe.

[22] McCabe C., Dimopoulos K., Pitcher A., Orchard E., Price L.C., Kempny A., Wort S.J. (2020). Chronic thromboembolic disease following pulmonary embolism: Time for a fresh look at old clot. Eur. Respir. J..

[23] Nakano Y., Adachi S., Nishiyama I., Yasuda K., Imai R., Yoshida M., Iwano S., Kondo T., Murohara T. (2022). Usefulness of a refined computed tomography imaging method to assess the prevalence of residual pulmonary thrombi in patients 1 year after acute pulmonary embolism: The Nagoya PE study. J. Thromb. Haemost..

[24] Huisman M.V., Klok F.A. (2015). Current challenges in diagnostic imaging of venous thromboembolism. Hematol. Am. Soc. Hematol. Educ. Program.

[25] Delcroix M., Torbicki A., Gopalan D., Sitbon O., Klok F.A., Lang I., Jenkins D., Kim N.H., Humbert M., Jais X. (2021). ERS statement on chronic thromboembolic pulmonary hypertension. Eur. Respir. J..

[26] Kovacs G., Bartolome S., Denton C.P., Gatzoulis M.A., Gu S., Khanna D., Badesch D., Montani D. (2024). Definition, classification and diagnosis of pulmonary hypertension. Eur. Respir. J..

[27] Kim N.H., D’Armini A.M., Delcroix M., Jaïs X., Jevnikar M., Madani M.M., Matsubara H., Palazzini M., Wiedenroth C.B., Simonneau G. (2024). Chronic thromboembolic pulmonary disease. Eur. Respir. J..

[28] Modarai B., Burnand K.G., Humphries J., Waltham M., Smith A. (2005). The role of neovascularisation in the resolution of venous thrombus. Thromb. Haemost..

[29] Mukhopadhyay S., Johnson T.A., Duru N., Buzza M.S., Pawar N.R., Sarkar R., Antalis T.M. (2019). Fibrinolysis and Inflammation in Venous Thrombus Resolution. Front. Immunol..

[30] Grau E., Moroz L.A. (1989). Fibrinolytic activity of normal human blood monocytes. Thromb. Res..

[31] Altmann J., Sharma S., Lang I.M. (2016). Advances in our understanding of mechanisms of venous thrombus resolution. Expert. Rev. Hematol..

[32] Wagenvoort C.A. (1995). Pathology of pulmonary thromboembolism. Chest.

[33] Kearon C., Akl E.A. (2014). Duration of anticoagulant therapy for deep vein thrombosis and pulmonary embolism. Blood.

[34] Morris T.A. (2013). Why acute pulmonary embolism becomes chronic thromboembolic pulmonary hypertension: Clinical and genetic insights. Curr. Opin. Pulm. Med..

[35] Karpov A.A., Vaulina D.D., Smirnov S.S., Moiseeva O.M., Galagudza M.M. (2022). Rodent models of pulmonary embolism and chronic thromboembolic pulmonary hypertension. Heliyon.

[36] Mansueto G., Costa D., Capasso E., Varavallo F., Brunitto G., Caserta R., Esposito S., Niola M., Sardu C., Marfella R. (2019). The dating of thrombus organization in cases of pulmonary embolism: An autopsy study. BMC Cardiovasc. Disord..

[37] Lang I.M., Pesavento R., Bonderman D., Yuan J.X. (2013). Risk factors and basic mechanisms of chronic thromboembolic pulmonary hypertension: A current understanding. Eur. Respir. J..

[38] Sista A.K., Klok F.A. (2018). Late outcomes of pulmonary embolism: The post-PE syndrome. Thromb. Res..

[39] Miniati M., Monti S., Bottai M., Scoscia E., Bauleo C., Tonelli L., Dainelli A., Giuntini C. (2006). Survival and restoration of pulmonary perfusion in a long-term follow-up of patients after acute pulmonary embolism. Medicine.

[40] Cosmi B., Nijkeuter M., Valentino M., Huisman M.V., Barozzi L., Palareti G. (2011). Residual emboli on lung perfusion scan or multidetector computed tomography after a first episode of acute pulmonary embolism. Intern. Emerg. Med..

[41] Planquette B., Ferré A., Peron J., Vial-Dupuy A., Pastre J., Mourin G., Emmerich J., Collignon M.-A., Meyer G., Sanchez O. (2016). Residual pulmonary vascular obstruction and recurrence after acute pulmonary embolism. A single center cohort study. Thromb. Res..

[42] Ma K.A., Kahn S.R., Akaberi A., Dennie C., Rush C., Granton J.T., Anderson D., Wells P.S., Rodger M.A., Solymoss S. (2018). Serial imaging after pulmonary embolism and correlation with functional limitation at 12 months: Results of the ELOPE study. Res. Pract. Thromb. Haemost..

[43] Jervan Ø., Dhayyat A., Gleditsch J., Haukeland-Parker S., Tavoly M., Klok F.A., Rashid D., Stavem K., Ghanima W., Steine K. (2023). Demographic, clinical, and echocardiographic factors associated with residual perfusion defects beyond six months after pulmonary embolism. Thromb. Res..

[44] The PIOPED Investigators (1990). Value of the ventilation/perfusion scan in acute pulmonary embolism. JAMA.

[45] Stevens S.M., Woller S.C., Kreuziger L.B., Bounameaux H., Doerschug K., Geersing G.J., Huisman M.V., Kearon C., King C.S., Knighton A.J. (2021). Antithrombotic Therapy for VTE Disease: Second Update of the CHEST Guideline and Expert Panel Report. Chest.

[46] https://www.nice.org.uk/guidance/ng158.

[47] Sardar P., Chatterjee S., Mukherjee D. (2013). Efficacy and safety of new oral anticoagulants for extended treatment of venous thromboembolism: Systematic review and meta-analyses of randomized controlled trials. Drugs.

[48] Weitz J.I., Lensing A.W., Prins M.H., Bauersachs R., Beyer-Westendorf J., Bounameaux H., Brighton T.A., Cohen A.T., Davidson B.L., Decousus H. (2017). Rivaroxaban or Aspirin for Extended Treatment of Venous Thromboembolism. N. Engl. J. Med..

[49] Agnelli G., Buller H.R., Cohen A., Curto M., Gallus A.S., Johnson M., Porcari A., Raskob G.E., Weitz J.I., AMPLIFY-EXT Investigators (2013). Apixaban for extended treatment of venous thromboembolism. N. Engl. J. Med..

[50] Van Es N., Coppens M., Schulman S., Middeldorp S., Büller H.R. (2014). Direct oral anticoagulants compared with vitamin K antagonists for acute venous thromboembolism: Evidence from phase 3 trials. Blood.

[51] Marik P.E., Cavallazzi R. (2015). Extended Anticoagulant and Aspirin Treatment for the Secondary Prevention of Thromboembolic Disease: A Systematic Review and Meta-Analysis. PLoS ONE.

[52] Bradbury C., Fletcher K., Sun Y., Heneghan C., Gardiner C., Roalfe A., Hardy P., McCahon D., Heritage G., Shackleford H. (2020). A randomised controlled trial of extended anticoagulation treatment versus standard treatment for the prevention of recurrent venous thromboembolism (VTE) and post-thrombotic syndrome in patients being treated for a first episode of unprovoked VTE (the ExACT study). Br. J. Haematol..

[53] Baglin T., Douketis J., Tosetto A., Marcucci M., Cushman M., Kyrle P., Palareti G., Poli D., Tait R.C., Iorio A. (2010). Does the clinical presentation and extent of venous thrombosis predict likelihood and type of recurrence? A patient level metaanalysis. J. Thromb. Haemost..

[54] Alhadad A., Miniati M., Alhadad H., Gottsäter A., Bajc M. (2012). The value of tomographic ventilation/perfusion scintigraphy (V/PSPECT) for follow-up and prediction of recurrence in pulmonary embolism. Thromb. Res..

[55] Kroft L.J.M., Erkens P.M.G., Douma R.A., Mos I.C.M., Jonkers G., Hovens M.M.C., Durian M.F., Cate H.T., Beenen L.F.M., Kamphuisen P.W. (2015). Prometheus Follow-Up Investigators. Thromboembolic resolution assessed by CT pulmonary angiography after treatment for acute pulmonary embolism. Thromb. Haemost..

[56] Becattini C., Giustozzi M., Cerdà P., Cimini L.A., Riera-Mestre A., Agnelli G. (2019). Risk of recurrent venous thromboembolism after acute pulmonary embolism: Role of residual pulmonary obstruction and persistent right ventricular dysfunction. A meta-analysis. J. Thromb. Haemost..

[57] Robin P., Le Pennec R., Eddy M., Sikora L., Le Roux P.Y., Carrier M., Couturaud F., Tromeur C., Planquette B., Sanchez O. (2023). Residual pulmonary vascular obstruction and recurrence after acute pulmonary embolism: A systematic review and meta-analysis of individual participant data. J. Thromb. Haemost..

[58] Barco S., Konstantinides S., Huisman M.V., Klok F.A. (2018). Diagnosis of recurrent venous thromboembolism. Thromb. Res..

[59] Chopard R., Genet B., Ecarnot F., Chatot M., Napporn G., Hyvert A., Didier-Petit K., Schiele F., Meneveau N. (2017). Detection of Residual Pulmonary Vascular Obstruction by Ventilation-Perfusion Lung Scan Late After a First Pulmonary Embolism. Am. J. Cardiol..

[60] Fauché A., Presles E., Sanchez O., Jaïs X., Le Mao R., Robin P., Pernod G., Bertoletti L., Jego P., Parent F. (2022). Frequency and predictors for chronic thromboembolic pulmonary hypertension after a first unprovoked pulmonary embolism: Results from PADIS studies. J. Thromb. Haemost..

[61] Klok F.A., van der Hulle T., den Exter P.L., Lankeit M., Huisman M.V., Konstantinides S. (2014). The post-PE syndrome: A new concept for chronic complications of pulmonary embolism. Blood Rev..

[62] Gharepapagh E., Rahimi F., Koohi A., Bakhshandeh H., Mousavi-Aghdas S.A., Sadeghipoor P., Fakhari A., Amirnia M., Javadrashid R., Rashidi F. (2023). Clot Burden As a Predictor of Chronic Thromboembolic Pulmonary Hypertension After Acute Pulmonary Embolism: A Cohort Study. Thorac. Res. Pract..

[63] Liu G., Wen J., Lv C., Liu M., Li M., Fang K., Fei J., Zhang N., Li X., Wang H. (2024). Development and validation of a Prediction Model for Chronic Thromboembolic Pulmonary Disease. Respir. Res..

[64] Tavoly M., Utne K.K., Jelsness-Jørgensen L.P., Wik H.S., Klok F.A., Sandset P.M., Ghanima W. (2016). Health-related quality of life after pulmonary embolism: A cross-sectional study. BMJ Open.

[65] Amato R.D., Ramírez Martín M.P. (2017). Prevalence and clinical predictors of persistent perfusion defects after acute pulmonary embolism. Eur. Respir. J..

[66] Jervan Ø., Parker S., Gleditsch J., Hansen K., Risberg M.A. (2022). Health-Related Quality of Life and Physical Capacity in Patients with Residual Perfusion Defects After Pulmonary Embolism [Poster Presentation]. https://www.eventscribe.net/2022/program/fsPopup.asp?Mode=presInfo&PresentationID=1078256.

[67] Cimini L.A., Luijten D., Barco S., Ghanima W., Jervan Ø., Kahn S.R., Konstantinides S., Lachant D., Nakano Y., Ninaber M. (2024). Pulmonary perfusion defects or residual vascular obstruction and persistent symptoms after pulmonary embolism: A systematic review and meta-analysis. ERJ Open Res..

